# Supplementary material for: Hippocampal neurons code individual episodic memories in humans
Source: Nat Hum Behav. 2023 Oct 5;7(11):1968–79. doi: 10.1038/s41562-023-01706-6 (PMC10663153; doi:10.1038/s41562-023-01706-6)
Supplement: Supplementary file 2 — Reporting Summary [file 41562_2023_1706_MOESM2_ESM.pdf]

nature portfolio

Corresponding author(s): Luca D. Kolibius, Simon Hanslmayr

Last updated by author(s): Aug 14, 2023

Reporting Summary

Nature Portfolio wishes to improve the reproducibility of the work that we publish. This form provides structure for consistency and transparency in reporting. For further information on Nature Portfolio policies, see our Editorial Policies and the Editorial Policy Checklist.

Statistics

For all statistical analyses, confirm that the following items are present in the figure legend, table legend, main text, or Methods section.

n/a	Confirmed	
☐	☒	The exact sample size (*n*) for each experimental group/condition, given as a discrete number and unit of measurement
☐	☒	A statement on whether measurements were taken from distinct samples or whether the same sample was measured repeatedly
☐	☒	The statistical test(s) used AND whether they are one- or two-sided<br>*Only common tests should be described solely by name; describe more complex techniques in the Methods section.*
☒	☐	A description of all covariates tested
☐	☒	A description of any assumptions or corrections, such as tests of normality and adjustment for multiple comparisons
☐	☒	A full description of the statistical parameters including central tendency (e.g. means) or other basic estimates (e.g. regression coefficient) AND variation (e.g. standard deviation) or associated estimates of uncertainty (e.g. confidence intervals)
☐	☒	For null hypothesis testing, the test statistic (e.g. *F*, *t*, *r*) with confidence intervals, effect sizes, degrees of freedom and *P* value noted<br>*Give P values as exact values whenever suitable.*
☒	☐	For Bayesian analysis, information on the choice of priors and Markov chain Monte Carlo settings
☐	☒	For hierarchical and complex designs, identification of the appropriate level for tests and full reporting of outcomes
☒	☐	Estimates of effect sizes (e.g. Cohen's *d*, Pearson's *r*), indicating how they were calculated

Our web collection on statistics for biologists contains articles on many of the points above.

Software and code

Policy information about availability of computer code

Data collection	All experiments were presented and analyzed using MATLAB 2015a-2018a (The Mathworks, USA) with Psychophysics Toolbox Version 3 (https://github.com/Psychtoolbox-3/Psychtoolbox-3). Intracranial data was recorded using the ATLAS setup running the Cheetah software v1.1.0 (Neuralynx INC, USA).
Data analysis	Data analysis was performed using MATLAB 2020a (The Mathworks, USA) using the Statistics and Machine Learning Toolbox (v11.7), FieldTrip (v20220403), SPM (v12), Wave_clus (v3). Electrode localization was done with the help of MRIcron (v1.0.20190902) and visualized using Surf Ice (v1.0.20201102).<br>Analysis code has been deposited on Figshare (https://figshare.com/s/12fcaf4069e972a5c362).

For manuscripts utilizing custom algorithms or software that are central to the research but not yet described in published literature, software must be made available to editors and reviewers. We strongly encourage code deposition in a community repository (e.g. GitHub). See the Nature Portfolio guidelines for submitting code & software for further information.

Data

Policy information about availability of data

All manuscripts must include a data availability statement. This statement should provide the following information, where applicable:

- Accession codes, unique identifiers, or web links for publicly available datasets
- A description of any restrictions on data availability
- For clinical datasets or third party data, please ensure that the statement adheres to our policy

Data and analysis code is currently available in a private repository on Figshare (https://figshare.com/s/12fcaf4069e972a5c362) and will be made publicly available upon publication. Raw data (.ncs files and MRIs) cannot be shared because they are not anonymized.

Human research participants

Policy information about studies involving human research participants and Sex and Gender in Research.

Reporting on sex and gender	Our first dataset consisted of 16 participants (7 female) while our second dataset consisted of 8 participants (4 female). Sex was not a variable of interest in our research.
Population characteristics	All patients had treatment resistant epilepsy and underwent surgery to localize epileptic foci. In our first dataset participants were on average 36.125 years old (26-53 years) while patients in the second dataset were on average 34.375 years old (19-58 years). Age has not been a variable of interest in our research.
Recruitment	Patients were admitted into the hospital for localization of epileptic foci using depth electrodes. Epilepsy patients were recruited by the local clinical teams through direct invitations to participate in the experiments. Patients received no componesation for their participation. Patients were not aware of the planned analyses or research questions. All patients gave informed consent and volunteered to participate while hospitalized.
Ethics oversight	Ethical approval was granted by the National Health Service Health Research Authority (15/WM/0219) and the Ethik-Kommission of the Friedrich-Alexander Universität Erlangen-Nürnberg (142_12 B). Informed consent was obtained in accordance with the Declaration of Helsinki.

Note that full information on the approval of the study protocol must also be provided in the manuscript.

Field-specific reporting

Please select the one below that is the best fit for your research. If you are not sure, read the appropriate sections before making your selection.

- [x] Life sciences
- [ ] Behavioural & social sciences
- [ ] Ecological, evolutionary & environmental sciences

For a reference copy of the document with all sections, see nature.com/documents/nr-reporting-summary-flat.pdf

Life sciences study design

All studies must disclose on these points even when the disclosure is negative.

Sample size	Our sample size is comparable to other published studies (Zheng et al., 2022 Nat Neurosc [N = 20]; Donoghue et al., 2023 Hippocampus [N = 5]; Kunz et al., 2021, Neuron [N = 15]). No sample-size calculation was performed.
Data exclusions	One patient was excluded due to poor/below chance memory performance. Another patient was excluded due to wrong filter settings during data acquisition leading to the loss of spike activity.
Replication	We here report findings from two independent datasets. The second dataset included a visual tuning task after a memory task. The memory task in the second experiment was largely the same memory association task as used before, but only consisted of one cue and one associate instead of two associate stimuli. We were able to replicate our original findings in the second dataset.
Randomization	All patients performed the same memory association task, so randomization was not applicable here. All covariates (age, sex, number of single neurons) are listed in the supplementary material.
Blinding	Patients were unaware of the hypotheses and to be performed data analyses while participating in the experiment. LDK and SH were aware of the hypothesis when analyzing the data. There was no group allocation.

Reporting for specific materials, systems and methods

We require information from authors about some types of materials, experimental systems and methods used in many studies. Here, indicate whether each material, system or method listed is relevant to your study. If you are not sure if a list item applies to your research, read the appropriate section before selecting a response.

Materials & experimental systems

n/a	Involved in the study
☒	☐ Antibodies
☒	☐ Eukaryotic cell lines
☒	☐ Palaeontology and archaeology
☒	☐ Animals and other organisms
☒	☐ Clinical data
☒	☐ Dual use research of concern

Methods

n/a	Involved in the study
☒	☐ ChIP-seq
☒	☐ Flow cytometry
☒	☐ MRI-based neuroimaging
